# Sub-arctic palsa degradation and the role of climatic drivers in the largest coherent palsa mire complex in Sweden (Vissátvuopmi), 1955–2016

**DOI:** 10.1038/s41598-020-65719-1

**Published:** 2020-06-02

**Authors:** Mats Olvmo, Björn Holmer, Sofia Thorsson, Heather Reese, Fredrik Lindberg

**Affiliations:** Department of Earth Sciences, University of Gothenburg, Box 460, SE 405 30, Gothenburg, Sweden

**Keywords:** Climate sciences, Environmental sciences

## Abstract

Substantial palsa degradation has occurred in Fennoscandia, which is considered to be driven by global climate change. Deeper understanding of the role of different climatic drivers on palsa decay, however, is lacking. We use meteorological data and aerial photographs from 1955 to 2016 to statistically identify the most important climatic drivers affecting changes in lateral-temporal palsa decay rates in the largest coherent palsa complex in Sweden, Vissátvuopmi. We show that wetter, warmer and shorter winters are the main causes of large and rapid changes in lateral-palsa extent since the mid-1950s. By analyzing meteorological data from the 1880s to present, we show that average annual temperature conditions have been unfavourable for palsas for more than a century and average annual precipitation conditions have been unfavourable since the 1940s. The decay rates have likely been amplified over the past 50-60 years, and in particular over the most recent decades, due to the combined effect of adverse air temperature and precipitation conditions. Palsa loss is expected to continue, most likely at a higher rate than today, with serious ecological impacts as a consequence.

## Introduction

**Figure 1:**
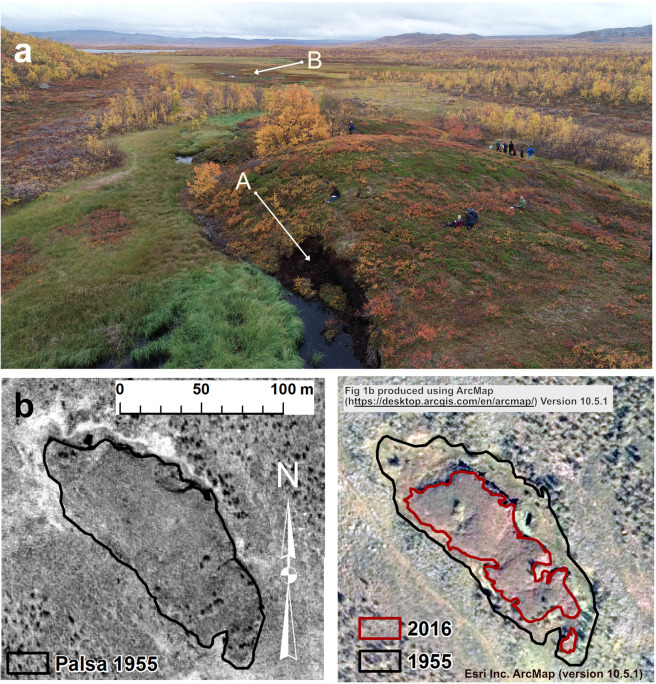
(**a**) Drone image showing part of the study area. In the foreground: the studied ridge-shaped palsa with recent peat-block erosion in the lower central part of the image (A). A minor palsa plateau (brownish colours at B) is seen beyond the ridge-shaped palsa. Photo by Mats Olvmo, Sept. 12 2019 at 9.20 AM, Location N 68.798, E 21.2104 looking southwards. (**b**) Extent and change in palsa area 1955 to 2016 of palsa shown in the foreground in Fig. 1a (Area C in Fig. 2c). During these 61 years more than half (54%) of the palsa area has decayed. B/W aerial image recorded 1955 July 28, Colour orthophoto from 2016 August 18. Data sources: Ortophoto, 1 m colour © Lantmäteriet (2016); Aerial photograph Lantmäteriet, 1955 (no copyright is claimed). Maps created by Mats Olvmo using ESRI ArcMap (https://desktop.arcgis.com/en/arcmap/) Version 10.5.1.

Palsas are peat mounds or plateaus with a perennially frozen (permafrost) core and formed primarily in subarctic wetland areas in the northern hemisphere (Fig. 1). Palsas represent marginal permafrost features and their distribution at the outer limit of the permafrost zone make them sensitive to climate variations^1–3^. The recognition of a cyclic development of palsas^4,5^ complicates the assessment of possible effects of climate change on palsa development, however, these effects may be detected by general trends towards either growth or decay^6^.

Over the past decades, substantial lateral palsa decay (i.e., degradation caused by peat block erosion, cracks in the peat surface and palsa collapse) has been reported in Fennoscandia with few signs of new palsa formation^1,7,8^. Thaw depths have also increased (e.g.^9,10^). The rate of lateral decay is accelerating^11^ and is expected to continue with a high risk of total palsa loss in Fennoscandia by the end of the 21^st^ century^12^. The ongoing palsa decay is considered to be driven by increasing air temperature and precipitation (e.g.,^7,10,13,14^), however, deeper understanding of the role that different climatic drivers exert on palsa decay rates is lacking.

## Results and discussion

### Changes in lateral palsa extent and decay rates from 1955 to 2016

**Figure 2:**
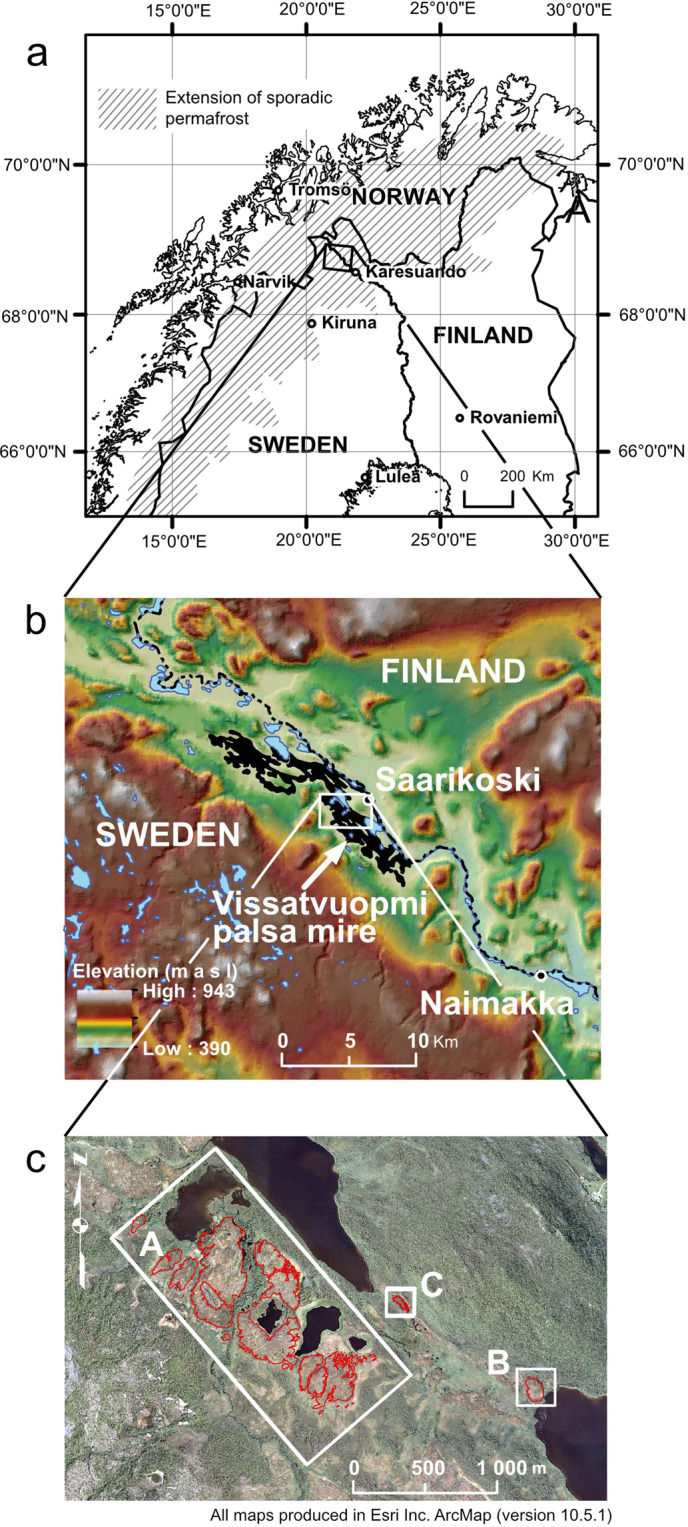
(**a**) Geographical setting of the study area with extension of sporadic permafrost in Fennoscandia adapted from Gisnås *et al*.^36^. Administrative boundaries based on data from EuroGeographics; UN-FAO © EuroGeographics for the administrative boundaries downloaded from https://ec.europa.eu/eurostat/web/gisco/geodata/reference-data/administrative-units-statistical-units/countries#countries16. Map produced by Mats Olvmo using ESRI ArcMap (https://desktop.arcgis.com/en/arcmap/) Version 10.5.1. (**b**) Extent of the Vissátvuopmi palsa mire complex shown in black. Data sources: ASTER GDEM v3 from NASA EOSDIS Land Processes DAAC. https://doi.org/10.5067/ASTER/AST14DEM.003; GSD-Översiktkartan © Lantmäteriet. Map compiled by Mats Olvmo using ESRI ArcMap (https://desktop.arcgis.com/en/arcmap/) Version 10.5.1. c) Colour orthophoto (2016 August 18) showing the mapped palsa areas, A) palsa plateau, B) dome-shaped palsa and C) ridge-shaped palsa. The outline of the identified palsa areas is marked with a red line. Data source: Ortophoto, 1 m colour © Lantmäteriet (2016). Map by Mats Olvmo using ESRI ArcMap (https://desktop.arcgis.com/en/arcmap/) Version 10.5.1.

Changes in lateral palsa extent in the Vissátvuopmi palsa complex (N 68°74′50″, E 21°11′30″; Fig. 2), the largest coherent palsa complex in Sweden, were mapped using manual interpretation of aerial photography-based orthophotos from 1955, 1963, 1994, 2010 and 2016 (Supplementary, Table 1).

**Figure 3:**
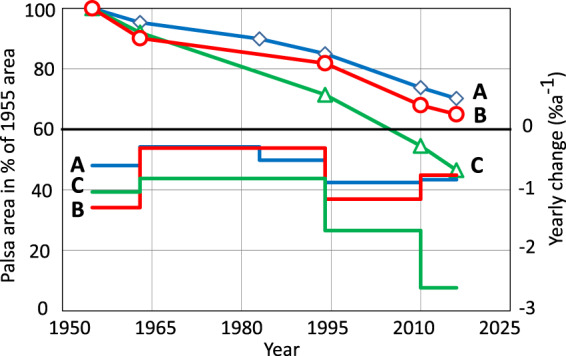
Changes in lateral palsa extent in three different areas in the Vissátvoupmi palsa complex between 1955 and 2016. Area A is dominated by palsa plateaus, Area B by dome-shaped palsas and Area C by ridge-shaped palsas.

The area dominated by palsa plateaus (Area A) has decreased in lateral area from 69.5 ha to 48.8 ha between 1955 and 2016, corresponding to a 30% lateral loss and an average annual decay rate of −0.58%a^−1^ (Fig. 3). The annual decay rate varied over the period, with the lowest average annual decay rate between 1963 and 1983 (−0.29%a^−1^) and the highest average annual decay rate between 1994 and 2010 (−0.88%a^−1^). The annual decay rate between 2010 and 2016 was −0.83%a^−1^, indicating a continued high degradation rate. This means that the average annual decay rate over the past decades (1994 to 2016) was twice that from the earlier period (1955 to 1994). The results are consistent with previous studies, showing substantial and varying palsa decay rates in Fennoscandia^1,7,8,11^.

The areas with dome-shaped (Area B) and ridge-shaped (Area C) palsas have experienced a higher decay rate than the area dominated by palsa plateaus (Fig. 3), which is in line with previous studies (e.g.,^11,14^). Over one-third (35%) of the dome-shaped palsa’s area and more than half (54%) of the ridge-shaped palsa´s area has decayed between 1955 and 2016, with an annual decay rate of −0.71%a^−1^ in Area B and −1.25%a^−1^ in Area C. This indicates that palsa morphology influences palsa decay rate. For instance, the height of the palsa may influence the decay rate as a higher palsa accumulates more snow on the leeward side due to snow drift, than a lower palsa. The thicker the snow depth, the less cold penetration there would be in winter and the more meltwater in spring and summer, leading to increased local permafrost thawing and block erosion. In addition, the higher palsa may be exposed to stronger winds and hence abrasion of the peat layer^15^. As a consequence, the peat may reach a critical level at which the insulating effect during the summer is lost. More studies are needed to better understand the relationship between palsa decay and palsa morphology.

Previous studies of lateral-temporal changes of palsa using aerial photos^11,14^ (see Supplement, Table 3) show higher decay rates than reported here. One possible explanation is that the examined palsas are smaller than in our study and thus the lateral and vertical extent of the frozen core are also smaller, resulting in a higher percentage melting of the core with the same amount of downward heat flux as for larger palsas. The fact that smaller palsas are more sensitive to enhanced downward heat flux than larger palsas is shown by the fact that small palsas can collapse within a few years^1,7,14^.

### Climate variations and change between 1955 and 2016

**Figure 4:**
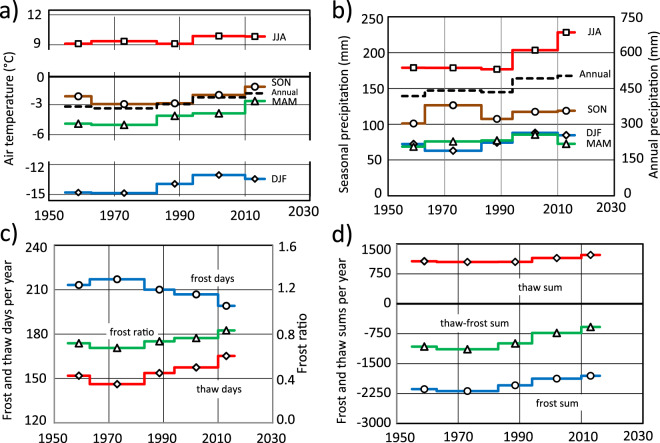
Seasonal (**a**) air temperature, (**b**) precipitation, (**c**) number of frost/thaw days and (**d**) frost and thaw sums in the study area from 1955–2016. Based on height-adjusted Karesuando data (**a, c** and **d**) and observed Karesuando data (**b**).

Average annual and seasonal air temperature, precipitation, number of frost and thaw days, and frost and thaw sums between 1955 and 2016 were derived from monthly air temperature and precipitation observations from two nearby meteorological stations (Karesuando and Naimakka, Fig. 2a,b). During the first half of the study period (1955–1994) there were small changes in air temperature (Fig. 4a). However, during the second half of the study period (1994–2016), the changes are obvious. Air temperature rose about 2 °C particularly in MAM (March-April-May) and SON (September-October-November) but less (0.8 °C) in JJA (June-July-August). As a consequence of increasing air temperature, the number of thaw days (average daily air temperature ≥ 0 °C) increased by 19 days (Fig. 4c). The thaw and frost sums (accumulated daily temperatures above/below 0 °C) both increased and as a result the net balance changed from −1100 to about −600 degree days (Fig. 4d).

During the second half of the study period, summer (JJA) precipitation increased by more than 50 mm. Precipitation in midwinter (DJF) also increased by just over 20 mm, which corresponds to a substantial amount of snow. Precipitation in MAM and SON showed no specific trend (Fig. 4b). Thus, there has been a shift in the heat balance towards a warmer, longer and moister thawing season, resulting in enhanced downward heat flux and thawing of permafrost. The winter has also become warmer and moister, resulting in increasing snowfall and snow depth, and in turn, enhanced snow cover insulation and decreased freezing. Due to snow drift as well as differences in number of days below zero between the study area and Karesuando, the impact of increased precipitation in the form of snow is uncertain.

### The role of different climatic drivers on lateral-temporal palsa decay

Linear regression models were used to analyse the role of different climatic drivers on lateral palsa decay in the larger plateau palsa area (Area A) and identify those of greatest importance. The linear models were evaluated using leave-one-out-cross-validation (LOOCV). The LOOCV uncertainty was quantified by the mean-square-error (MSE).

**Figure 5:**
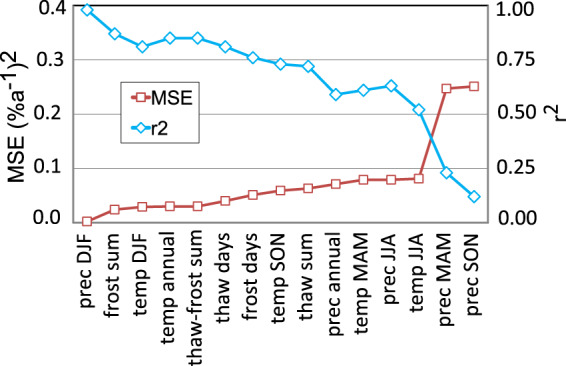
Mean-square-error (MSE) and corresponding explained variance (r^2^) for the regressions models.

The model with the lowest MSE was that for precipitation in DJF, followed by frost sum and air temperature in DJF, showing that wetter, warmer and shorter winters are the main cause of the large and rapid change in palsa extent over the past 60 years (Fig. 5 and Table 1). However, it is also clear that changes in summer conditions (i.e., increased air temperatures and precipitation) had an impact on the decay rates. All models are statistically significant to at least the 90% level (p < 0.1), except for the spring and autumn precipitation models.

**Table 1:** Regressions of palsa decay rates in relation to climatological variables, equilibrium points (*x* when *y* = 0) and anomalies compared with equilibrium points. Based on data 1955–2016, *n* = 5. LOOCV Leave-One-Out-Cross-Validation.

variable	r^2^	significance	slope	intercept	equilibrium point	LOOCV equilibrium point range		average 1987–2016	average-equilibrium point anomaly
						min	max		
Air temp annual (°C)	0.85	**	−0.413	−1.67	**−4.0**	−4.8	−3.9	−2.1	**1.9**
Air temp SON (°C)	0.73	**	−0.365	−1.39	−3.8	−5.1	−3.4	−1.8	2.0
Air temp DJF (°C)	0.81	**	−0.283	−4.51	−15.9	−17.1	−15.5	−12.9	3.0
Air temp MAM (°C)	0.61	*	−0.256	−1.63	−6.4	−9.2	−5.7	−3.5	2.9
Air temp JJA (°C)	0.52	*	−0.544	4.67	8.6	7.7	8.9	9.8	1.2
Precip. annual (mm)	0.59	*	−0.006	2.24	**363**	299	394	481	**118**
Precip. SON (mm)	0.12	No sig.	—	—	—	—	—	—	—
Precip. DJF (mm)	0.98	**	−0.024	1.19	50.4	49.0	51.3	85	35
Precip. MAM (mm)	0.23	No sig.	—	—	—	—	—	—	—
Precip. JJA (mm)	0.63	*	−0.011	1.59	139	99	155	202	63
Frost days	0.76	**	0.038	−8.52	226	223	237	206	−20
Thaw days	0.81	**	−0.036	4.99	137	129	141	158	21
Frost sum (°days)	0.87	**	−0.002	−3.75	−2408	−2584	−2359	− 1871	537
Thaw sum (°days)	0.72	**	−0.003	3.22	927	804	972	1137	210
Frost-Thaw sum (°days)	0.85	**	−0.001	−1.63	−1462	−1743	−1393	−734	728

**Significant on 95% level, *significant on 90% level.

### Climate conditions required for palsa development

**Figure 6:**
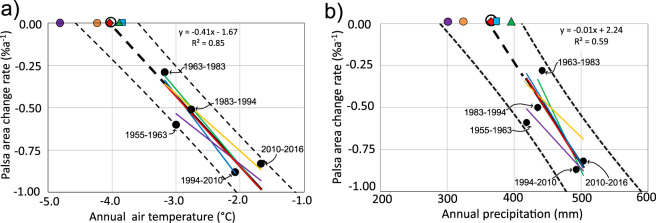
Annual palsa degradation vs (**a**) annual air temperature and (**b**) annual precipitation. Annual decay rates were calculated from the averages of 6- to 16-year periods (see labels in (**a**)). Thick dashed black line is the regression line for all observations, coloured lines are regression lines from each cross-validation (LOOCV), black circle is equilibrium point for all observations, coloured markers are equilibrium points due to cross-validation, and dashed curves are 95% confidence limits for all the observations’ regression line.

The “equilibrium point” is the value of a climatic driver where the annual freezing and thawing of a palsa is assumed to be in balance (i.e., when the average annual decay rate is zero). It is estimated by calculating the point where the regression line of the palsa degradation rate crosses the x axis at y = 0 for the different regression models. The annual air temperature and precipitation equilibrium points are estimated to be −4.0 °C (LOOCV range =  [−4.8, −3.9]; r^2^  =  0.85; 95% confidence interval CI  =  [ − 4.6, −3.6]) and 363 mm (LOOCV range =  [299, 394]; r^2^ = 0.59, CI =  [290, 411]), respectively (Table 1 and Fig. 6). Thus, cross-validation with LOOCV and 95% confidence intervals give ranges of the equilibrium points that are of similar magnitude although not exactly overlapping. The equilibrium point for annual air temperature is within the range of optimum areal occurrence of palsas in comparison with the 1961-90 climate^16^. However, the study found that optimum areal occurrence was associated with annual precipitation less than 300 mm, which is at the lower end of our equilibrium intervals.

Over the last 30-year period of the study (1987–2016) the average annual air temperature was −2.1 °C and annual precipitation was 481 mm, i.e., there was an anomaly of 1.9 °C and 118 mm, respectively (Table 1). In fact, all climate variables except the number of frost days show large anomalies at the end of the study period compared to the equilibrium points (Table 1). This implies that adverse climate conditions (air temperature and precipitation) for palsas currently prevail in the study area.

### Climate conditions for palsa development from 1880 to 2016

**Figure 7:**
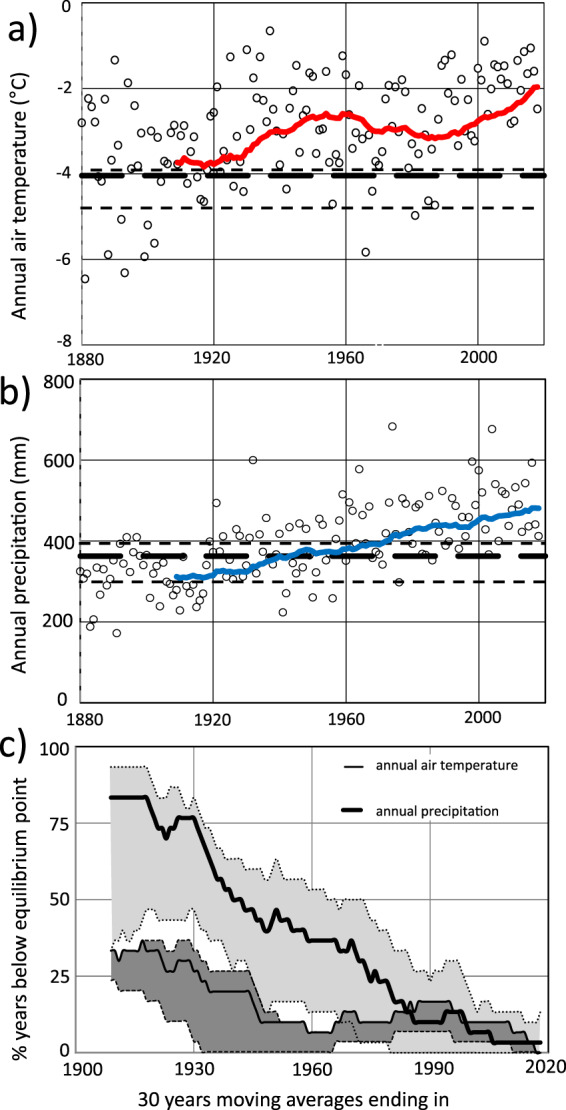
Equilibrium points compared to (**a**) annual air temperature and (**b**) precipitation since 1880 (annual and 30 years’ moving average). Thick dashed line is the equilibrium point based on all five intervals, thin dashed line is range of equilibrium points according to cross-validation (LOOCV), and (**c**) frequencies of years below equilibrium points for air temperature and precipitation. Shaded areas show the range of frequencies due to the cross-validation. Air temperatures are from Karesuando data that are height-adjusted to the palsa study area while precipitation amounts are unadjusted Karesuando data.

Average annual air temperature and precipitation from 1880 to 2016 and 30-year running averages were derived from monthly air temperature and precipitation observations from Karesuando meteorological station (Fig. 2a). The average annual air temperature has increased by 1.8 °C since the 1880s and the 30-year running average has been above the air temperature equilibrium point (−4.0 °C) since the beginning of the 20^th^ century; it is presently 1.9 °C above the equilibrium point (Fig. 7a). Between 1880 and 1910 large interannual variations with a number of years well below the equilibrium point were common, and favourable to palsa growth. The years between 1910 and the mid-1960s were characterised by increasing average annual air temperature and a large number of years well above the equilibrium point, favourable to palsa decay. This relatively warm period was followed by a slightly cooler period (from about 1965 to the late 1980s) with some years well below the equilibrium point. From the late-1980s and onwards the average annual air temperature has increased rapidly, with no years below the equilibrium point and many years well above the equilibrium point.

The general change in air temperature pattern in the study area fits well with the change in air temperatures in the circumpolar zonal band 65–75°N^17^. However, in our study area the anomalies are larger between the 1930s and 1940s as well as during the last decades, which may be explained by the ice-albedo effect that has an effect up to hundreds of kilometres from the coast^17^.

Like the average annual air temperature, the average annual precipitation has increased since the 1880s and in 2018 it was about 100 mm above the precipitation equilibrium point (363 mm; Fig. 7b). Between 1880 and 1920, the 30-year running average was below the precipitation equilibrium point (363 mm) and years with average annual precipitation well below the equilibrium point were common. In the 1940s the 30-year running average exceeded the equilibrium point and since the mid-1980s there have not been any years in which the average annual precipitation has been below the equilibrium point.

Figure 7c shows the 30-year running average frequency of years below the air temperature and precipitation equilibrium points from 1910 and onwards. As shown, the frequency has decreased from 33% to 0%, and 83% to 4%, respectively. The decrease in frequency of years below the air temperature equilibrium point was interrupted by an increase between 1960s and 1990s. This may explain the relatively slow palsa decay observed in the aerial photos between 1963 and 1994 (Fig. 3).

To summarise, the average annual air temperature conditions have been unfavourable for palsas for more than a century. Since the 1940s both air temperature and precipitation conditions have been unfavourable and today climate conditions favouring new palsa formation no longer exist. This implies that the palsa decay observed in the aerial photographs from 1955 and onward is a result of combined unfavourable air temperature and precipitation conditions. The fact that the air temperature conditions were unfavourable for palsas also in the first part of 20^th^ century (before aerial photographs were available for this area) can be confirmed by signs of palsa decay, such as the large number of thermokarst lakes, in the aerial photographs from 1955. However, the palsa decay rate was probably lower then than after 1955 as only the average annual air temperature was unfavourable for palsas.

If the decay rate continues at the same rate as during the last two decades (−0.9%a^−1^), half of the Vissátvuopmi palsa area may be lost by the end of the 21^st^century. The fact that smaller palsas are more sensitive to enhanced downward heat flux than larger palsas implies that the lateral decay rate is expected to increase as the palsas become smaller. Thus, it is most likely that future decay rates will be higher than today due to a combination of increased temperature, precipitation, and the palsas’ geomorphology and size. Decay rates of 2%a^−1^ and even higher rates have been observed in other areas^11,14^ (Supplementary Table 3), which may partly be explained by palsa size since the palsas studied in these areas are generally smaller than the mapped area in Vissátvuopmi.

The air temperature equilibrium point presented here (−4 °C) is −3 to −1 °C lower than previously suggested^16,18–20^ to delimit palsa areas. This may be explained by the fact that the air temperature conditions required to balance freezing and thawing of palsas was based on “present day” conditions, not taking into account that palsas have likely been in a state of continuous decay for more than a century. The poor correlation between palsa distribution in Northern Norway and air temperature during different periods of the 20th century has been discussed by Åhman^21^ who found that air temperatures in 1931–1960 were too high to match the palsa distribution, concluding that the poor correlation was due to inertia in how the palsas reacted to climate variations. With increasing temperatures in the future, the areas where palsas can exist will diminish. In a modelling approach with seven GCMs^22^ it was found that in all but one scenario all suitable regions for palsas would be lost at the end of the 21st century, compared to the climate 1961–90. However, the calculations are based on the assumption that there is a balance between climate and palsa distribution, while the present study shows a considerable imbalance between palsa area and climate in the period 1963–1994, which almost covers 1961–90, as well as the whole study period 1955–2016.

### Implications and further studies

The results imply that the palsas in Vissátvuopmi are highly threatened and have most likely been in a degradation phase since the early 20^th^ century. Given global warming trends, the climate conditions required for palsa development no longer exist in the study area and a total palsa loss in this area is expected to occur over a relatively short time scale, with serious impacts on hydrology, flora, fauna and humans, including changes in human livelihoods in these regions^23,24^. Vissátvuopmi is situated in an area with traditional reindeer herding that requires grazing areas and an infrastructure that enables the reindeer and herders to move between different areas. Permafrost thawing in general may have serious direct and indirect impacts on traditional reindeer herding practices^25^. Wetter conditions make it more difficult for both reindeer and reindeer herders to move between different grazing areas and can even cause animals to die by being trapped in thermokarst ponds, for example. Furthermore, grazing patterns are indirectly affected by the significant change in plant communities caused by thawing and subsidence of permafrost.

In addition, subarctic peat lands hold more than 30% of the global soil C store^26^ and as permafrost melts, greenhouse gases (e.g., methane) are released, leading to a negative feedback^24,27–29^. Direct observations show very high annual losses of carbon (>5%)^30^.

This study contributes to a better understanding of the influence of changing climate on the vulnerable palsa mire environment, however, further studies are needed. Previous studies have focused primarily on lateral changes of palsas for the simple reason that aerial photographs are easily available. However, permafrost depth and vertical-temporal degradation of palsa (i.e., degradation caused by thinning of the permafrost) have been less studied^31–33^. New geospatial technologies can provide three-dimensional data to map and monitor vertical- and volume-temporal change in palsas. These technologies include high-resolution digital surface models from photogrammetric image-matching of drone-based aerial images, as well as digital terrain models from airborne LiDAR or interferometric synthetic aperture radar data (e.g.,^34^). Further studies using these data should be applied for frequent and more accurate estimation of palsa and permafrost decay rates, particularly during the coming decades when more rapid palsa loss is expected.

## Methods

### Site description

The study area is located at approximately N 68°74′50″, E 21°11′30″ ca 2 km WSW of the settlement Saarikoski on the Swedish-Finnish border (Fig. 2). It comprises 51 ha corresponding to c. 20% of the Vissátvuopmi palsa complex, which is the largest coherent palsa complex in Sweden located in the core area of palsa distribution^18^. The studied part of the mire is located at an altitude between 440 and 445 m a s l. and is dominated by low (1–1.5 m) palsa plateaus (Area A), as well as dome-shaped (Area B) and ridge-shaped palsas (Area C) rising 3–4 m above the surrounding fen surface. The study area has been visited by the authors on several occasions since 1994.

### Data

Monthly air temperature and precipitation from Karesuando (SMHI station number 192830/192840, WHO station number 2–80; Fig. 2) and Naimakka (SMHI station number 19190/191910; Fig. 2) were used to calculate annual and seasonal air temperature, precipitation, number of frost and thaw days, and frost and thaw sum. The Karesuando meteorological station is situated in the broad, flat valley bottom 65 km southeast of the study area. The station was established in 1879 and has since moved five times, however all times within a distance of some hundreds of meters at an elevation between 327–333 m.a.s.l. The Naimakka meteorological station is situated on a headland in the valley 17 km southeast of the study area (at 403 m.a.s.l.). The station was established in 1944 and became automatic in 1996. The vertical air temperature gradient between the two stations was used to estimate the air temperature in the study area. The gradient ranged from around 0 °C/100 m in December to February to −1.7 °C/100 m in June. This yearly cycle of the temperature gradient is similar to observations in southern Norway^35^.

In 2007 a meteorological station (SMHI station number 194940) was established in Saarikoski measuring precipitation on the shore of the Könkämä River, 2 km east of the study area. Precipitation data from this site were compared to the data from Karesuando and Naimakka, but no systematic differences were observed; precipitation data from Karesuando were therefore used without corrections for the palsa area. Frost and thaw days as well as frost and thaw sums were obtained through linear interpolation of monthly air temperatures.

### Aerial and orthophoto image interpretation

Changes in palsa extent were mapped in the three different subareas (Fig. 2, Areas A-C) using visual interpretation of orthorectified aerial photographs delivered by the Swedish National Land Survey (Supplementary, Table 2). In addition, analogue copies of aerial photographs from 1963 and 1994 were used for stereoscopic visualization in order to facilitate the interpretation, especially in situations where interpretation was difficult. All areas are covered by aerial photos from the years 1955, 1963, 1994, 2010 and 2016, while photos from 1959 and 1983 covered only areas C and A, respectively (Supplementary, Table 2). The aerial photos used are considered to be of good quality. Disturbances that could emanate from e.g., clouds or photo processing are absent. The resolution of the 1994 photos is somewhat lower than the rest of the photos, but considered acceptable. Aerial photos from 1981 are available, but were not used since the resolution was considered to be too low. The aerial photos are recorded at different times during the growing season, which may cause some error, however, since the vegetation on palsas is dominated by low shrubs, lichens and mosses–differing distinctly from the vegetation in the surrounding wet fen areas–the contrast between palsas and fen is quite distinct in both B/W and colour images.

By using these aerial photographs, the boundaries of the coherent palsa plateau, dome- and ridge-shaped palsas for each observation year were digitized and the changes in lateral extent of the palsas between the different observation periods determined. To ensure a consistent interpretation, the same person delineated the palsa boundaries for each sub-area. To estimate the accuracy and subjective bias of the mapping procedure, two interpreters independently mapped Area A. This comparison yielded a difference of lateral extension of 2 and 7% (Supplementary Table 3). For the statistics in this paper, we have used the interpreter with the most field experience in the study area (i.e., interpreter 1).

In all interpretations, there were some small areas which indicated lateral increases between time periods. Since this increase was just “slivers” in the delineated data that did not persist through the years, it was most likely due to quality differences between the images, small errors in georeferencing, or to differences among the interpreters in marking the exact position of the palsa border. Considering that our analysis supports that palsa degradation started before 1955 (shown by the large number of thermokarst lakes in the aerial photographs from 1955) and the reasons stated above, we made the assumption that growth had not occurred during the investigated period. This was done by stepping backwards from 2016 and incorporating any degradation of the palsa extent as compared to the previous images (2010, 1994, 1963 and 1955).

### Statistical analysis

Palsa extents for each image year were calculated and used for quantitative analysis of decay rates. To account for difference in the time interval between aerial photos, the average annual decay rate (AADR; %a^−1^) was calculated using

1 $${\rm{AADR}}=\left({\left(\frac{{{\rm{A}}}_{{\rm{start}}}}{{{\rm{A}}}_{{\rm{stop}}}}\right)}^{\frac{1}{{{\rm{Y}}}_{{\rm{start}}}-{{\rm{Y}}}_{{\rm{stop}}}}-1}\right)\times 100$$

where A_start_ and A_stop_ are the palsa extent at year Y_start_ and Y_stop_, respectively.

Linear regression models were used to analyze the role of different climatic drivers on lateral palsa decay in Area A and to statistically identify the climatic drivers of greatest importance for the changes in lateral-temporal palsa decay. The climate variables are average values for the same time periods as the observed yearly palsa decay rate in Fig. 3. Since the length of the time period differs, the average values are weighted according to the number of years in each time interval. A 95% confidence interval was calculated for the regression lines.

The linear regression models were also evaluated using leave-one-out-cross-validation (LOOCV), which successively leaves out one sample from a training data set and estimates models based on the remaining *n*−1 samples (*n* = 5). The LOOCV uncertainty was quantified by the mean-square-error (MSE) using

2 $$RMS{E}_{LOOCV}=\sqrt{\frac{1}{n}\mathop{\sum }\limits_{i=1}^{n}MS{E}_{i}}$$

where *n* is the number of samples for the regression analysis (5) and MSE is calculated using

3 $$MS{E}_{i}={({y}_{i}-{{ {\hat{y}} }}_{i})}^{2}$$

where *y*_*i*_ is the palsa decay rate for year *i*, and *ŷ*_*i*_ is the LOOCV-estimated rate of palsa decay for year *i*.

## Supplementary information

Supplementary Information.
