# Supplementary material for: Sub-arctic palsa degradation and the role of climatic drivers in the largest coherent palsa mire complex in Sweden (Vissátvuopmi), 1955–2016
Source: Sci Rep. 2020 Jun 2;10:8937. doi: 10.1038/s41598-020-65719-1 (PMC7265444; doi:10.1038/s41598-020-65719-1)
Supplement: Supplementary file 1 — Supplementary Information. [file 41598_2020_65719_MOESM1_ESM.pdf]

Sub-arctic palsa mire degradation and the role of climatic drivers in the largest coherent palsa complex in Sweden (Vissátvuopmi), 1955-2016

Mats Olvmo[1]*, Björn Holmer[1], Sofia Thorsson[1], Heather Reese[1] & Fredrik Lindberg[1]

[1] Department of Earth Sciences, University of Gothenburg, Box 460, SE 405 30 Gothenburg, Sweden.
Correspondence and requests for materials should be addressed to M.O. (email: matso@gvc.gu.se).

Supplementary

Table 1. Key information about the aerial photos used in the study. GSD is the Ground Surface Distance, i.e. the distance between the centres of two adjacent pixels measured on the ground. A) palsa plateau, B) dome-shaped palsa and C) ridge-shaped palsa.

Date of registration	Cover (area)	Source	Image scale	GSD (m)	Film type
1955 July 28	A, B, C	Swedish Land Survey	1: 29 000	0.64	Panchromatic (B/W)
1959 July 20	C	Swedish Land Survey	1: 30 000	0.68	Panchromatic (B/W)
1963 August 1	A, B, C	Swedish Land Survey	1: 30 000	0.49	Panchromatic (B/W)
1983 June 28	A	Swedish Land Survey	1: 15 000	0.61	Panchromatic (B/W)
1994 July 28	A, B, C	Swedish Land Survey	1: 30 000	0.99	Panchromatic (B/W)
2010 August 18	A, B, C	Swedish Land Survey	ND	0.5	Digital IR-colour
2016 August 18	A, B, C	Swedish Land Survey	ND	0.5	Digital RGB

Table 2. Comparison of interpretations of lateral extension of palsas in area A between the three independent interpreters.

Year	Interpreter 1	Interpreter 2	Interpreter 3	I 1/2	I 1/3	I 2/3
**1955**	648018 m^2		636203 m^2		1.02	
**1963**	626667 m^2	608182 m^2	605398 m^2	1.03	1.04	1.00
**1983**	607977 m^2	596127 m^2	598396 m^2	1.02	1.02	1.00
**1994**	574462 m^2	564806 m^2	564614 m^2	1.02	1.02	
**2010**	502555 m^2	517443 m^2	486629 m^2	0.97	1.03	1.06
**2016**	487824 m^2		471473 m^2		1.03	

Table 3. Calculated yearly palsa decay rates (%a^{-1}) in Borge et al. (2017) and Mamet et al. (2017)

		Palsa areas (ha)							Change (%a^{-1})					
Mamet et al. (2017)														
HF	year	*1944*	*1974*	*1981*	*2010*	*2016*		interval	*1944-1974*	*1974-1981*	*1981-2010*	*2010-2016*		
	area	0.35	0.28	0.25	0.13	0.10		rate	-0.73	-1.67	-2.32	-4.14		
BP	year	*1944*	*1972*	*1981*	*2010*	*2016*		interval	*1944-1974*	*1972-1981*	*f1981-2010*	*2010-2016*		
	area	0.62	0.55	0.52	0.35	0.30		rate	-0.43	-0.60	-1.40	-2.47		
D6	year	*1949*	*1972*	*1981*	*2013*	*2016*		interval	*1949-1974*	*1974-1981*	*1981-2013*	*2013-2016*		
	area	0.39	0.21	0.17	0.09	0.08		rate	-2.60	-2.67	-1.91	-4.66		
D2	year	*1949*	*1972*	*1981*	*2013*	*2016*		interval	*1944-1972*	*1972-1981*	*1981-2013*	*2013-2016*		
	area	0.25	0.17	0.15	0.10	0.08		rate	-1.65	-0.90	-1.40	-7.33		
GF	year	*1944*	*1949*	*1974*	*1981*	*2010*	*2016*	interval	*1944-1949*	*1949-1974*	*1974-1981*	*1981-2010*	*2010-2016*	
	area	1.21	0.86	0.45	0.23	0.05	0.04	rate	-6.50	-2.58	-8.86	-5.18	-4.80	
Borge et al. (2017)														
Karlebotn	year	*1957*	*2005/08*					year	*1957-2005*	*1957-2008*				
	area	217	100					rate	-1.60	-1.51				
Laxelv	year	*1959*	*2008*					year	*1959-2008*					
	area	95	49					rate	-1.34					
Goatheluoppal	year	*1958*	*1980*	*2003*	*2012*			year	*1958-1980*	*1980-2003*	*2003-2012*	*1958-2012*		
	area	50	35	21	15			rate	-1.64	-2.13	-4.04	-2.25		
Suossjavri	year	*1956/59*	*1982*	*2003*	*2011*			year	*1956-1982*	*1959-82*	*1982-2003*	*2003-2011*	*1956-2011*	*1959-2011*
all palsas	area	74	65	55	50			rate	-0.50	-0.57	-0.75	-1.40	-0.73	-0.77
4 largest plateaus								rate					-0.30	-0.31
domes and small plateaus								rate					-1.18	-1.25
